# Comparison of peripapillary vessel density between preperimetric and perimetric glaucoma evaluated by OCT-angiography

**DOI:** 10.1371/journal.pone.0184297

**Published:** 2017-08-31

**Authors:** Si Bum Kim, Eun Jung Lee, Jong Chul Han, Changwon Kee

**Affiliations:** Department of Ophthalmology, Samsung Medical Center, Sungkyunkwan University School of Medicine, Seoul, Korea; Bascom Palmer Eye Institute, UNITED STATES

## Abstract

**Purpose:**

To determine peripapillary vessel density in eyes with perimetric glaucoma (PG) or preperimetric glaucoma (PPG) compared to normal controls using optical coherence tomography-angiography (OCT-A).

**Methods:**

We recruited 13 patients with unilateral perimetric normal-tension glaucoma (NTG) and fellow preperimetric NTG showing only inferotemporal retinal nerve fiber layer (RNFL) defect in red-free RNFL photography in both eyes. We also enrolled 9 healthy controls. Using OCT-A, radial peripapillary capillary densities at inferotemporal and superotemporal regions were evaluated. Paired comparison of peripapillary vessel density was performed for PG eye, PPG eye, and normal eye.

**Results:**

A total of 26 eyes of the 13 patients with unilateral PG and fellow PPG eyes and 18 eyes of 9 normal controls were analyzed. Vessel densities at the whole peripapillary region and inferotemporal region in PG eyes were significantly lower than those in PPG eyes (*P* = 0.001, *P*<0.001, respectively). Comparison between PPG and normal eyes showed no significant difference in the whole peripapillary region or the inferotemporal region (*P* = 0.654, *P* = 0.174, respectively). There was no significant (*P* = 0.288) difference in vessel density at superotemporal region among the three types of eyes (PG eye, PPG eye, and normal eye).

**Conclusion:**

PPG eyes and normal eyes were found to have the similar densities of peripapillary microvasculature at the area with nerve fiber layer defect, whereas in PG eye, there was a significant decrease in vessel density at the area of RNFL thinning. This provides evidence that microvascular compromise in the retina might be a secondary change to nerve fiber layer degeneration in the pathogenesis of NTG.

## Introduction

Normal tension glaucoma (NTG) is a multi-factorial optic neuropathy characterized by damage to the retinal nerve fiber layer (RNFL) and the optic nerve head associated with corresponding visual field (VF) defect, although office-hour intraocular pressure (IOP) does not exceed normal range in NTG. The pathophysiology of NTG remains largely undetermined. It has been hypothesized that abnormal ocular blood flow (OBF) is involved in the pathogenesis of this disease. A number of evidences have suggested that vascular factors play significant roles in the development of NTG[1–4].

The association between OBF and glaucoma has been investigated using several imaging methods, including fluorescein angiography[5–9], Heidelberg retina flowmeter[10], color Doppler imaging[11], laser speckle flowgraphy[12,13], and laser Doppler velocimetry[14]. Optical coherence tomography angiography (OCT-A) is a new imaging device that can characterize vasculature in various retinal layers, providing quantitative assessment of the microcirculation in the optic nerve head (ONH) and peripapillary region[15–18].

Based on OCT-A, it has been reported that microvasculatures in ONH and peripapillary retina, especially at radial peripapillary capillaries (RPC) layer, are reduced in glaucomatous eyes. In ONH and RPC layers, blood flow and vessel densities calculated by OCT-A are significantly lower in glaucomatous eyes than those in healthy eyes, and these findings showed spatial concordance with nerve fiber layer thinning or visual field defects[16–29]. However, the sequential relationship between microvascular reduction and RNFL degeneration has not been identified yet. Which event arises first is still controversial [30–36].

We hypothesized that microvascular changes might occur after RNFL degeneration if vessel changes were secondary changes according to glaucomatous RNFL changes. Preperimetric glaucoma (PPG) was defined as the presence of characteristic glaucomatous changes in the optic disc and increased vulnerability to damage in the RNFL without the presence of VF defects detectible with standard automated perimetry. PPG could be considered as temporally previous stage of perimetric glaucoma (PG)[37,38]. Therefore, comparison of peripapillary vessel density between PG and PPG might give key to the sequential relationship of microvascular reduction and RNFL degeneration. Thus, the objective of this study was to compare peripapillary vessel densities of perimetric glaucomatous eyes (***PG eyes***), preperimetric glaucomatous eyes (***PPG eyes***), and ***normal eyes*** using OCT-A.

## Methods

### Study population

This study was a cross-sectional study performed from June 1, 2016 to June 30, 2016 at Samsung Medical Center, South Korea. Patients with bilateral NTG who had a unilateral PG eye and a fellow PPG eye were recruited. Healthy volunteers were also recruited. Research protocols were approved by the Institutional Review Board of Samsung Medical Center. The study was performed in accordance with the tenets of the Declaration of Helsinki. Written informed consent was obtained from each participant.

The inclusion criteria for **PG eyes** were:(1) IOP < 21 mmHg without history of elevated office-hour IOP, (2) glaucomatous changes of the optic disc, including neuroretinal rim thinning, notching, cupping, and disc hemorrhage; (3) RNFL defect and/or thinning on red-free RNFL photography images and/or spectral-domain OCT; (4) consistent glaucomatous pattern on both qualifying Humphrey Swedish Interactive Threshold Algorithm (SITA) 30–2 VF tests with pattern standard deviation outside the normal limits (*P*< 0.05), glaucomatous hemifield test results outside normal limits, or the presence of a cluster of 3 or more adjacent non-edge points in typical glaucomatous locations that did not cross the horizontal meridian, all of which were depressed on pattern deviation plot at *P*< 0.05 level. One of which was depressed at *P*< 0.01 level on at least two consecutive plots.

The inclusion criteria for **PPG eyes** were as follows: (1) glaucomatous structural and IOP findings as stated above in criteria for PG eyes, (2) normal VF results.

The inclusion criteria for control eyes were as follows: (1) no evidence of retinal pathology or glaucoma; (2) IOP <21 mmHg without history of elevated IOP; (3) an open angle on gonioscopy; (4) normal appearing ONH and RNFL; and (5) normal findings of VF test.

When OCT-A exams were performed, one topical glaucoma medication (0.5% betaxolol or 0.005% latanoprost) was used for all PG and PPG eyes. None of normal eyes received any topical medications.

Exclusion criteria were as follows: (1) best-corrected visual acuity worse than 20/40; (2) a narrow anterior chamber angle in gonioscopic examination; (3) retinal and neurologic diseases that could affect VF test results; (4) a history of intraocular surgery; (5) VF mean deviation (MD) less than -15.0 decibels (dB); (6) refractive error greater than ± 6.0 diopters (D).

Both eyes of glaucomatous patients and normal volunteers were included in this study.

### Clinical examination

All subjects underwent comprehensive ophthalmologic examinations, including best-corrected visual acuity assessment, refractive error measurement by manual refraction, slit-lamp biomicroscopy, IOP measurement with Goldmann applanation tonometry, gonioscopic examination, ultrasound pachymetry (Tomey SP-3000, Tomey Ltd., Nagoya, Japan), dilated fundus examination with simultaneous stereophotography of the optic disc and red-free RNFL photography, Humphrey SITA VF tests using central 30–2 Humphrey Field Analyzer (HFA model 640 or model 740; Humphrey Instruments Inc., San Leandro, CA, USA), and spectral-domain OCT (Carl Zeiss Meditec Inc., Dublin, California, USA).

### OCT-A data acquisition and processing

OCT-A (Optovue Inc, Fremont, California, USA) provides a noninvasive OCT-based method for visualizing vascular structures of the retina. It uses 840 nm light source with an A-scan rate of 70,000 scans/s and a bandwidth of 50 nm. Each of acquired optic disc cubes consisted of 304 clusters of two repeat B-scans containing 304 A-scans each. A split-spectrum amplitude-decorrelation angiography (SSADA) algorithm was employed to improve the signal-to-noise ratio by splitting the spectrum to generate multiple repeat OCT frames from the two original repeat OCT frames[18].

OCT-A can characterize vascular information at various user-defined retinal layers as a vessel density map. It is quantitatively expressed as vessel density (%). Vessel density was automatically calculated as the proportion of measured area occupied by flowing blood vessels defined as pixels having decorrelation values. It was acquired with the SSADA algorithm above the threshold level.

### Calculation of peripapillary vessel density

**Fig 1:**
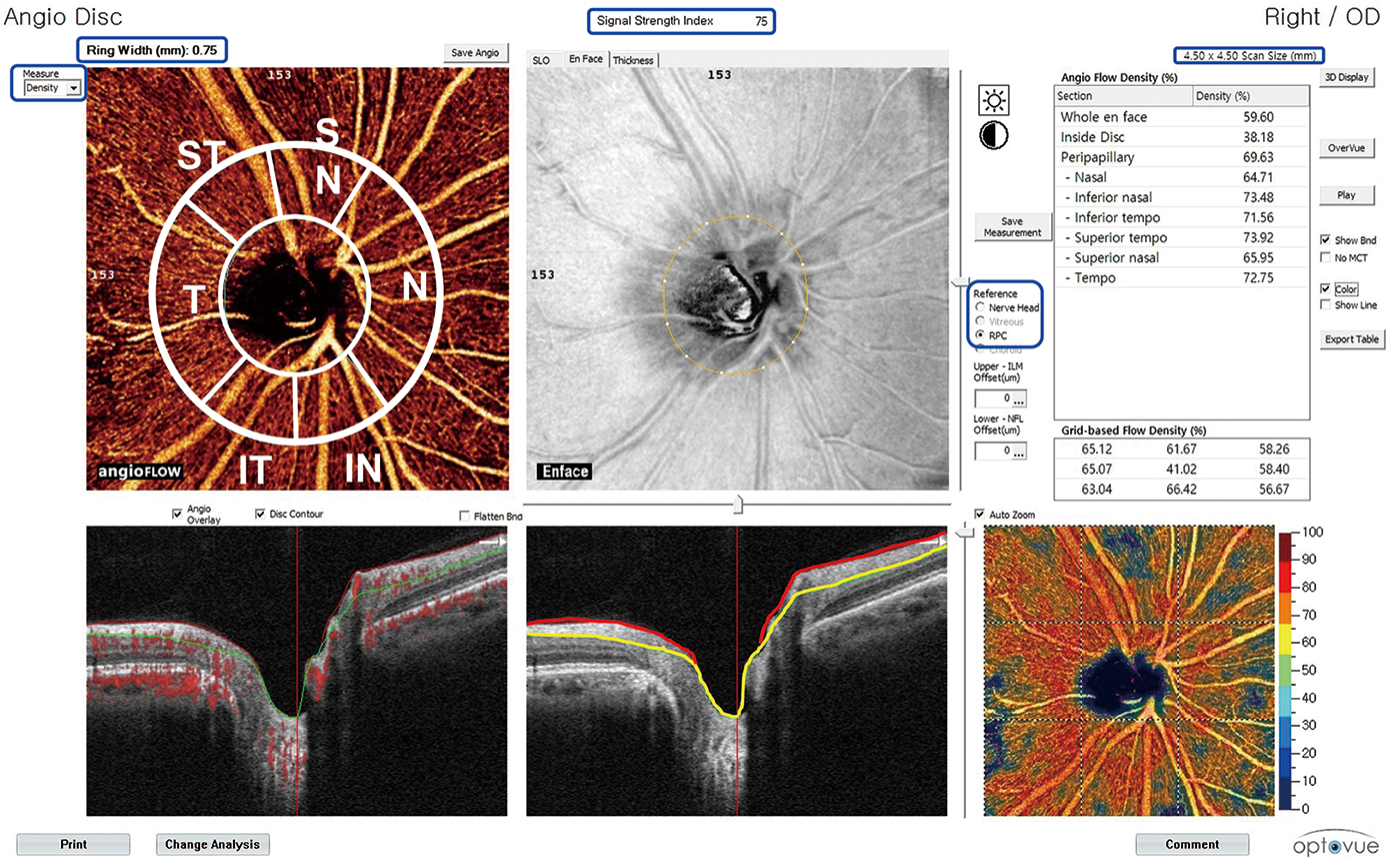
Analysis view of optical coherence tomography angiography. *Blue box*: Scan size is 4.50 x 4.50 mm and Ring Width on angioflow view is 0.75 mm (750μm). The signal strength index is “75”. Measurement mode is “density”. Reference line is “RPC (radial peripapillary capillaries)”. *White line* on angioflow view; Sectorial division of peripapillary region including nasal, inferonasal, inferotemporal, superotemporal, superonasal, and temporal regions was performed using Garway-Heath regionalization. The measured vessel densities of each region are presented in the right chart (green box) as %. *Scattered red dots* on B-scan view(left) indicates the detection of blood movement (angioflow). On right B-scan view, *red line* indicates internal limiting membrane while *yellow line* indicates RNFL posterior boundary using standard OCT-A software (version 2015.1.0.90). Red line to yellow line is the RPC layer.

In this study, vessel density in peripapillary RNFL was analyzed for images with a 4.5 X 4.5 mm field of view centered on the optic disc. Vessel density within the RNFL was measured in RPC layer as programmed setting from internal limiting membrane to RNFL posterior boundary using the standard OCT-A software (version 2015.1.0.90). Measurements were obtained for nine areas (Fig 1). Inside disc vessel density was measured for the region inside the optic disc boundary. Whole en face vessel density was measured for the entire 4.5 X 4.5 mm image while whole peripapillary vessel density was calculated for the region of 750 μm-wide elliptical annulus extending from the optic disc boundary. Sectorial division of peripapillary region including nasal, inferonasal, inferotemporal, superotemporal, superonasal, and temporal area was performed using Garway-Heath regionalization[39]. Among vessel densities calculated at total nine areas, those at whole peripapillary region, inferotemporal region (with RNFL defect), and superotemporal region (without RNFL defect) were used to compare vessel densites at areas with or without RNFL defect.

Additionally, vessel densities of superotemporal and inferotemporal choroidal layers were analyzed. Because this OCT-A software on mode of vessel density viewer could not provide vessel density of the choroidal layer, the vessel densities of choroid were calculated by manually shifting temporal reference line below the retinal pigment epithelium.

Poor quality images were excluded from analysis. Poor quality images had the following characteristics: (1) signal strength index of less than 48, (2) poor clarity, (3) residual motion artifacts visible as irregular vessel pattern or disc boundary on the enface angiogram, (4) local weak signal, or (5) RNFL segmentation errors. The location of the disc margin was reviewed for accuracy and adjusted manually when necessary.

### Statistical analysis

All statistical analyses were executed using SPSS Version 23 software (IBM Corp, Armonk, New York, USA), SAS Version 9.4 (SAS Institute, Cary, NC), and R 3.2.5 (Vienna, Austria, http://www.R-project.org/).

Wilcoxon signed-rank test was performed to compare ages. Fisher’s exact test was performed to compare sex distribution between glaucomatous patients and normal volunteers. Differences in IOP, central corneal thickness, spherical equivalent, VF mean deviation, pattern standard deviation, and vessel density among PG, PPG, and control eyes were evaluated using Kruskal-Wallis test. All comparisons were corrected with post hoc test. In post hoc analysis, significance of differences between groups was determined after Bonferroni correction. To adjust within-subject variation of statistical data that contained both eyes of each subject, generalized estimating equation was used for comparison among the three eye groups[40].

All data are presented as means ± standard deviations (SD) of the means. For all statistical analyses, *P*<0.05 was considered statistically significant.

## Results

Among 25 subjects (14 patients with a unilateral PG eye and a fellow PPG eye, 11 normal volunteers), 13 patients and 9 normal volunteers were included in the final analysis. One patient and two volunteers were excluded from analysis due to poor quality of OCT-A imaging.

Demographic and clinical characteristics of patients with NTG are shown in Table 1. The mean age of the control group was significantly smaller than that of patients with NTG (35.0 ±10.7 years vs. 55.3 ±10.6 years, *P*<0.001). However, sex distribution was not significantly different between the two groups (Male/Female: 7/6 vs. 5/4, *P* = 1.000). There were no statistically significant differences in IOP, central corneal thickness, or refractive errors among groups. VF parameters in PG eyes were significantly lower than those in PPG or normal eyes.

**Table 1:** Demographic and clinical characteristics of normal-tension glaucoma (NTG) and controls.

	PG eyes	PPG eyes	Control eyes	*P*
Age (y)	55.3 ±10.6		35.0 ±10.7	<0.001*
Sex (male/female)	7/6		5/4	1.000†
Intraocular pressure, mmHg	14.0 ±2.8	15.7 ±2.4	14.8 ±3.5	0.234‡
Central corneal thickness, μm	531.2 ±36.2	547.3 ±37.8	540.0 ±25.3	0.274‡
Refractive errors (Diopter)	-2.40 ±2.46	-1.82 ±2.04	-1.38 ±1.78	0.410‡
Number of using antiglaucoma eyedrop	1	1	0	-
Visual field MD, dB	-4.91 ±4.36	-0.52 ±1.99	-0.33 ±1.04	0.001‡
Visual field PSD, dB	9.02 ±4.80	2.45 ±0.93	1.98 ±1.11	<0.001‡

PG eyes: eyes with perimetric NTG, PPG eyes: eyes with preperimetric NTG

MD: mean deviation; dB: decibel; PSD: pattern standard deviation.

*P-value by Wilcoxon signed-rank test

†P- value by Fisher exact test

‡P value by Kruskal-Wallis test.

**Fig 2:**
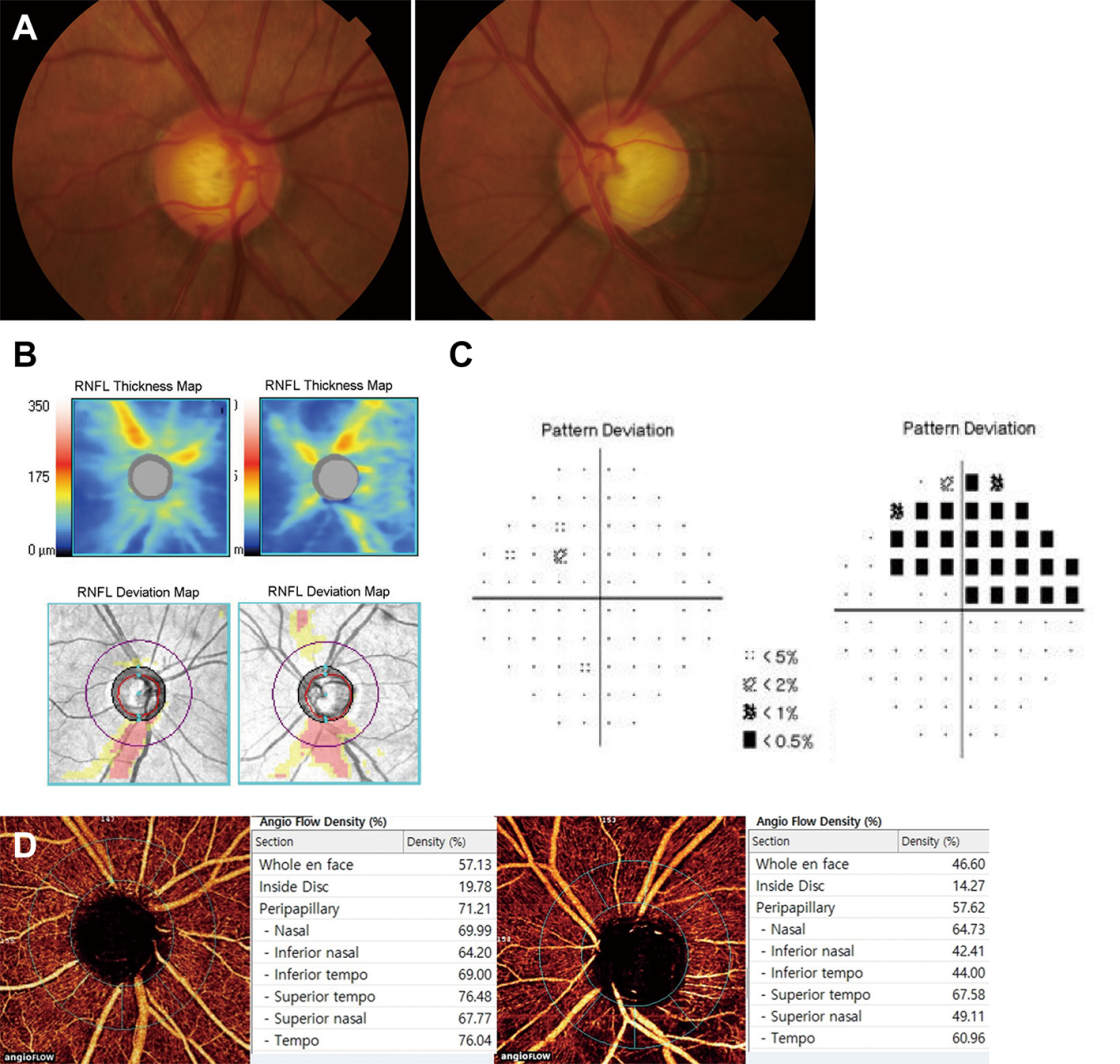
A representative case of unilateral PG eye (left eye) and fellow PPG eye (right eye). The patient was a 64-year-old man who was referred to the glaucoma clinic on annual examination. Treated intraocular pressure was 14 mmHg for both eyes. (A, B) Color disc photography and retinal nerve fiber layer (RNFL) thickness map and deviation map on spectral-domain OCT showing inferotemporal RNFL defect with optic disc hemorrhage in the right eye and notching in the left eye. (C) Visual field tests showing superior arcuate scotoma in the left eye. The right eye showed no abnormality. (D) Angioflow views are acquired from internal limiting membrane to RNFL posterior boundary. Based on gross observation, the right eye showed no drop-out of peripapillary vasculatures. However, the left eye showed wedge-shaped focal vascular reduction in the same area as RNFL defect. On angioflow density, inferotemporal vessel density in right eye was higher than that in left eye (69.00% versus 44.00%).

Only inferotemporal RNFL defect was observed in all included glaucomatous eyes. Vessel density of the RPC layer at the whole peripapillary region was significantly lower in PG eyes than that in PPG or control eyes (57.9% vs. 64.6%, *P* = 0.001; 57.9% vs. 63.2%, *P* = 0.004). Vessel density at the inferotemporal region with RNFL defect was also significantly lower in PG eyes than that in PPG or control eyes (48.3% vs.64.7%, 48.3% vs.68.4%, both *P*<0.001). PPG and control eyes did not show any significant difference in vessel densities in the whole peripapillary region or the inferotemporal region (64.6% vs. 63.2%, *P* = 0.654; 64.7% vs. 68.4%, *P* = 0.174). There was no significant difference in vessel densities at the superotemporal region without RNFL defect among the three groups of eyes (64.8% vs. 65.9% vs. 67.7%, *P* = 0.288) (Table 2). A representative case is demonstrated in Fig 2. On OCT-A angioflow views, PG eye showed wedge-shaped focal vascular reduction in the same area as RNFL defect with decreased RPC vessel density (44.0%). However, PPG eye showed no drop-out of peripapillary vasculature with higher RPC vessel density (69.0%) than PG eye.

**Table 2:** Comparison of vessel densities of the radial peripapillary capillaries layer of perimetric glaucomatous (PG) eyes, fellow preperimetric glaucomatous (PPG) eyes, and control eyes.

	PG eyes (A)	PPG eyes (B)	Control eyes (C)	P*	Post Hoc		
					A vs B	A vs C	B vs C
Whole Peripapillary (%)	57.9 ±6.24	64.6 ±5.22	63.2±4.11	0.001	0.001	0.004	0.654
Inferotemporal (%)	48.3 ±8.99	64.7 ±5.42	68.4 ±5.41	<0.001	<0.001	<0.001	0.174
Superotemporal (%)	64.8 ±7.44	65.9 ±7.78	67.7 ±4.01	0.288	0.265	0.052	0.258

*P-value by Kruskal-Wallis test.

**Fig 3:**
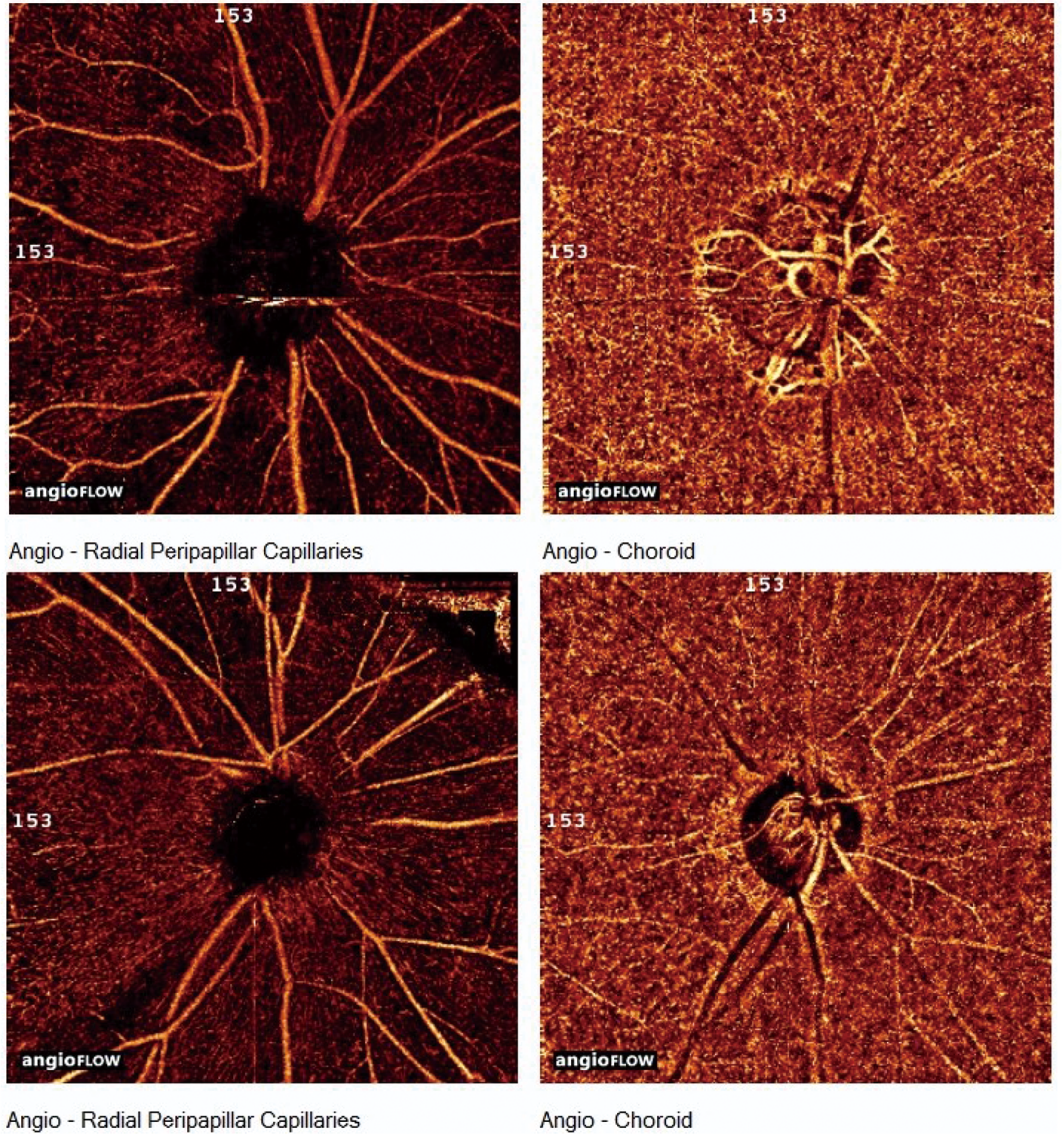
The view of angioflow at radial peripapillary capiallaries (RPC) and choroidal layer in each perimetric eye of two normal-tension glaucomatous patients (top and bottom row). Angioflow views on RPC layer (left column) showed wedge-shaped focal vascular reduction in the inferotemporal area. At the same location, angioflow views on choroidal layer (right column) showed no vascular reduction.

The vasculature and vessel densities of choroidal layer are shown in Fig 3 and Table 3. There was no focal reduction of choroidal vasculature observed grossly not only in PPG and control eyes, but also in PG eyes with wedge shaped vascular reduction of RPC layer on OCT-A angioflow view.

**Table 3:** Comparison of vessel densities of the choroidal layer of perimetric glaucomatous (PG) eyes, fellow preperimetric glaucomatous (PPG) eyes, and control eyes.

	PG eyes	PPG eyes	Control eyes	P*
Inferotemporal, (%)	69.4 ±4.43	69.0 ±5.12	71.3 ±8.18	0.386
Superotemporal, (%)	71.1 ±6.11	69.6 ±5.89	70.4 ±7.08	0.189

*P-value by Kruskal-Wallis test.

## Discussion

In the current study, RPC vessel density at the area of RNFL defect in PPG eyes was significantly higher than that in PG eyes. However, it was not significantly different from that in normal eyes.

Recently, several studies on ocular microvasculature, especially at peripapillary retina, have been reported. Liu et al.[17] first reported that peripapillary vessel density in glaucomatous eyes was decreased compared to normal eyes. Since then, several studies have shown differences in RPC vessel densities between normal and glaucomatous eyes, with decreased RPC vessel densities in glaucomatous eyes showing spatial concordance with RNFL defect[17,19,22,23,26–29]. These studies have also reported that RPC vessel density in glaucoma suspect is significantly higher than that in glaucomatous eyes. Furthermore, several studies have reported that there is no significant difference in vessel densities between glaucoma suspect and normal eyes[22,26,29]. However, these previous studies included only neuroretinal rim change without RNFL defect or RNFL thinning on OCT without considering RNFL change in glaucoma suspect. These earlier stages of glaucomatous eyes have less severity than those used in the current study. Furthermore, most of these studies except the studies of Akagi et al.[19] and Rao et al.[27] have compared vessel densities at whole peripapillary region, but not at sectorial division.

As stated above, there are several reports about decreased vessel density (microvascular reduction) at the area with RNFL degeneration. However, there has been no evidence about the sequential relationship between these two events. Recently, we have suggested the possibility that microvascular changes in the retina are secondary to nerve damage[41]. The following findings support such speculation: 1) Capillary compromise in the retina was wedge-shaped, not with geographic pattern, demonstrating ischemic capillary nonperfusion; 2) Vascular events as a primary cause of structural damages in the retina could not explain why they were damaged selectively in the superotemporal and the inferotemporal area; 3) Non-glaucomatous fellow eyes in unilateral NTG demonstrated thinner RNFL than normal eyes without showing decrease in retinal capillary densities compared to normal controls.

Therefore, the current study focused on PPG to obtain more evidence for this speculation. PPG was defined as the presence of characteristic glaucomatous changes in the optic disc and increased vulnerability to damage in the retinal nerve fiber layer without the presence of VF defects detectible with standard automated perimetry. Recent study of Jeong et al.[37] have reported the long-term clinical course of preperimetric NTG (71 eyes) for more than 4.5 years. They found that 41eyes (57.7%) showed progression in either structural or functional evaluation while perimetric glaucoma developed in 19 eyes (26.8%). In another study by Kim et al.[38], glaucoma progression was detected in 72 eyes (56.7%) of 127 PPG eyes with a follow-up of more than 5 years. A total of 29 eyes (22.8%) had VF deterioration.

PPG could be considered as temporally previous stage of PG. Therefore, comparison of peripapillary vessel density between PG and PPG might give key to the sequential relationship of microvascular reduction and RNFL degeneration.

In the current study, vessel densities of the RPC layer with or without RNFL defect were compared among PG, PPG, and normal eyes. The RPC vessel density at inferotemporal region with RNFL defect was significantly lower in PG eyes than that in PPG eyes. However, PPG and normal eyes showed no significant deference in vessel density at the same region. At superotemporal region without RNFL defect, there was no significant difference in vessel density among the 3 types of eyes. These results demonstrated that microvasculature of RPC layer in PPG eyes, even with RNFL defect, was as much intact as normal eyes. Therefore, we could speculate that RNFL degeneration might have occurred but peripapillary retinal vascular change did not occur yet or changed at a mild degree. In this sense, microvascular changes in the retina might have occurred after RNFL damage in the pathogenesis of NTG.

Akagi et al.[19] have evaluated microvascular density in glaucomatous eyes with hemifield VF defects and compared the RNFL thickness. Their results showed that the reduction in circumpapillary RNFL thickness was significant not only at the corresponding location of VF defects, but also at the noncorresponding location, while the reduction in the vessel density was limited to the corresponding location[19]. They suggested that peripapillary microvascular reduction might have occurred after RNFL thinning[19]. Their suggestion is in line with our conclusion.

The strong point of current study was that the comparison of vessel density was targeted at subjects who had unilateral PPG eye and fellow PG eye. Therefore, definite comparison was possible, although the number of samples was relatively small. Another strong point of the study was that we excluded advanced or severe stage of glaucoma through VF mean deviations. Therefore, it was possible to perform sectoral comparison, although such comparison might be difficult if there was diffuse RNFL defect. Additionally, we selected eyes with only inferotemoral change of neuroretinal rim and RNFL without superotemoral change. Therefore, we could compare the regions with and without RNFL defect, respectively.

When the choroidal vasculature was observed grossly, there was no focal reduction of choroidal vasculature. No difference in vessel density among the three categories of eyes unrelated to RNFL defect was found. These results indicate that the choroidal vasculature might not be affected in glaucoma. However, only peripapillary choroid was observed in this study. In addition, the vessel density at the choroid was calculated by manually shifting the reference line in OCT-A density viewer. Therefore, further studies are needed to measure the choroidal blood flow around ONH with programmed calculation.

This study has several limitations. First, we included both eyes of each patient and normal volunteer to compensate the small number of samples. Under this condition, within-subject variation should be considered. Therefore, we used generalized estimating equation[40] for comparison among the three types of eyes. Second, the age of normal volunteers was significantly younger than glacomatous patients. However, the younger distribution of age of normal volunteers actually strongly supports our conclusion. Younger eyes would be healthier with more nerve fibers, hence more vasculatures. Therefore, it is unlikely that our results would be biased due to deviated age range of normal volunteers. Third, this was a cross sectional study with a single observation at one specific point of follow-up period. Long-term variations should be investigated in the future. Fourth, we only analyzed peripapillary retinal vasculatures, not ONH vasculatures. In addition, OCT-A software-version 2015.1.0.90-was used in this study. It did not provide calculation for vessel density inside ONH. However, it has been reported that interferometric fringe washout effects from large blood vessels with fast flow rates can interfere with accurate measurement of ONH flow[42]. Therefore, this study concentrated on peripapillary microvasculatures. Recently, Rao et al.[27] have reported that ONH vessel density has significantly lower diagnostic abilities in open angle glaucoma than peripapillary vessel density and that the variability of ONH vessel density might be related to physiological variations generally seen in ONH with respect to disc size, shape, tilt, position of central retinal vessels, and so on.

In conclusion, vessel density at the area with RNFL defect in PPG eyes was found to be higher than that in PG eyes but similar to that in normal eyes. Results of this study presented new evidence supporting that microvascular change in the retina might be secondary to RNFL degeneration. This is not a denial on vascular or ischemic pathogenesis of glaucoma. Separately from inside-ONH causes that lead to RGC loss, we could identify that peripapillary vascular change in the retina is a secondary event. Longitudinal investigations would be beneficial in order to apply these findings to help monitoring and treatment of patients in the clinical field. In addition, further studies on ischemic or mechanical changes inside and around ONH are warranted.

## Supporting information

S1 Fig Age distribution of the study population. (JPG)

S2 Fig Distribution of whole peripapillary vessel densities. (JPG)

S3 Fig Distribution of inferotemporal peripapillary vessel densities. (JPG)

S4 Fig Distribution of superotemporal peripapillary vessel densities. (JPG)
