# Supplementary figures and images for: Comparison of peripapillary vessel density between preperimetric and perimetric glaucoma evaluated by OCT-angiography
Source: PLoS One. 2017 Aug 31;12(8):e0184297. doi: 10.1371/journal.pone.0184297 (PMC5578657; doi:10.1371/journal.pone.0184297)

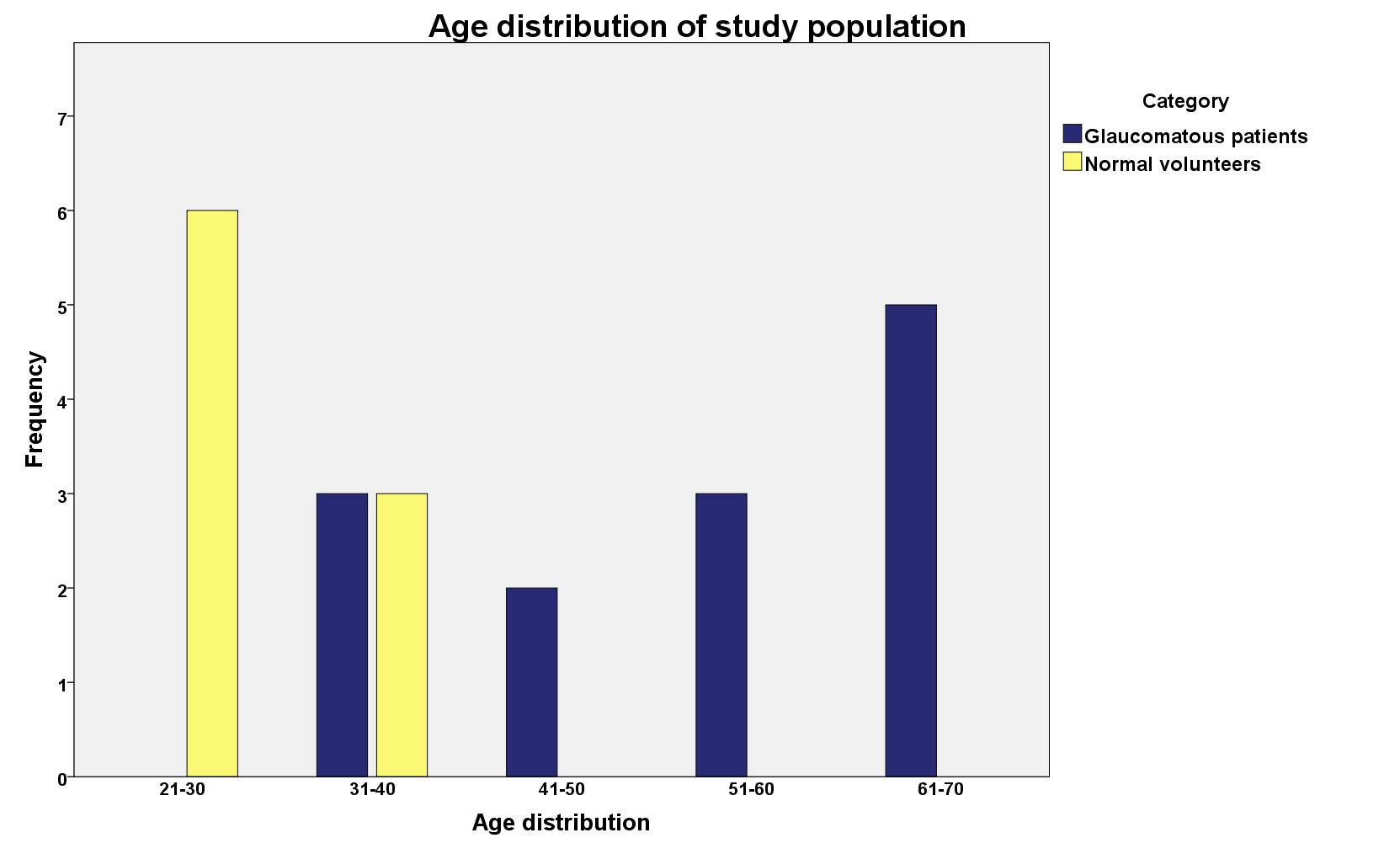

Supplement: S1 Fig — (JPG) [file pone.0184297.s001.jpg]

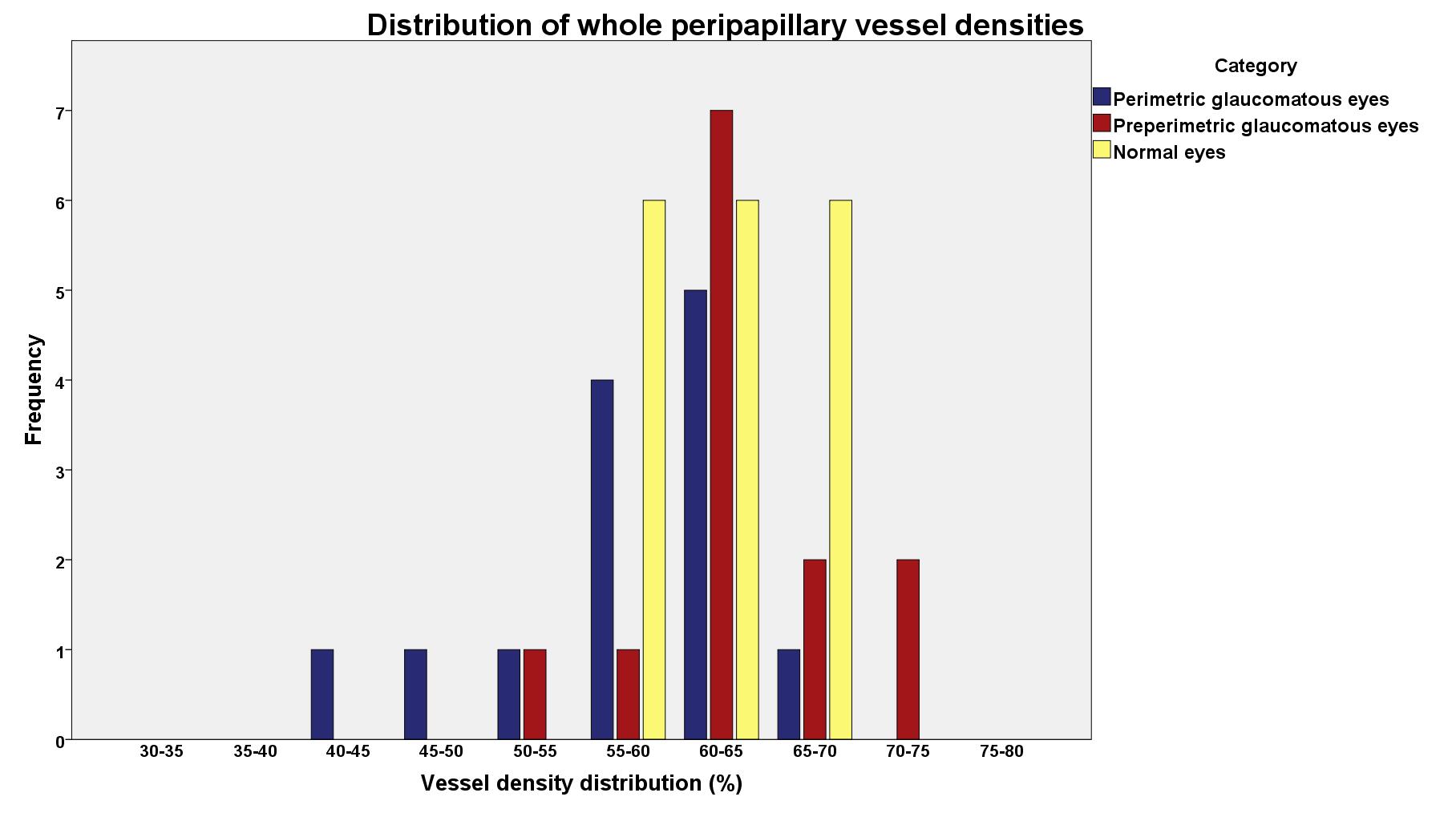

Supplement: S2 Fig — (JPG) [file pone.0184297.s002.jpg]

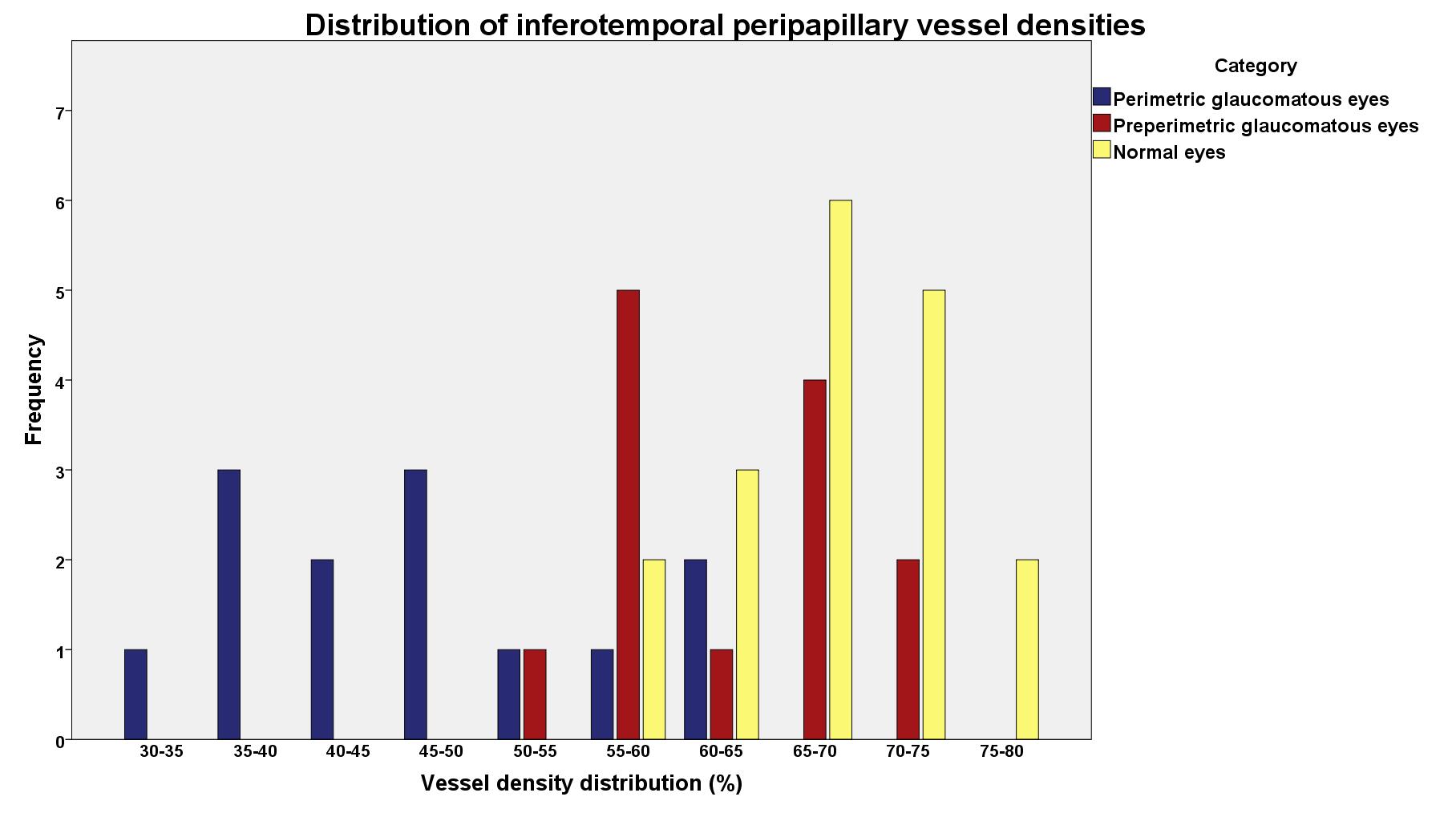

Supplement: S3 Fig — (JPG) [file pone.0184297.s003.jpg]

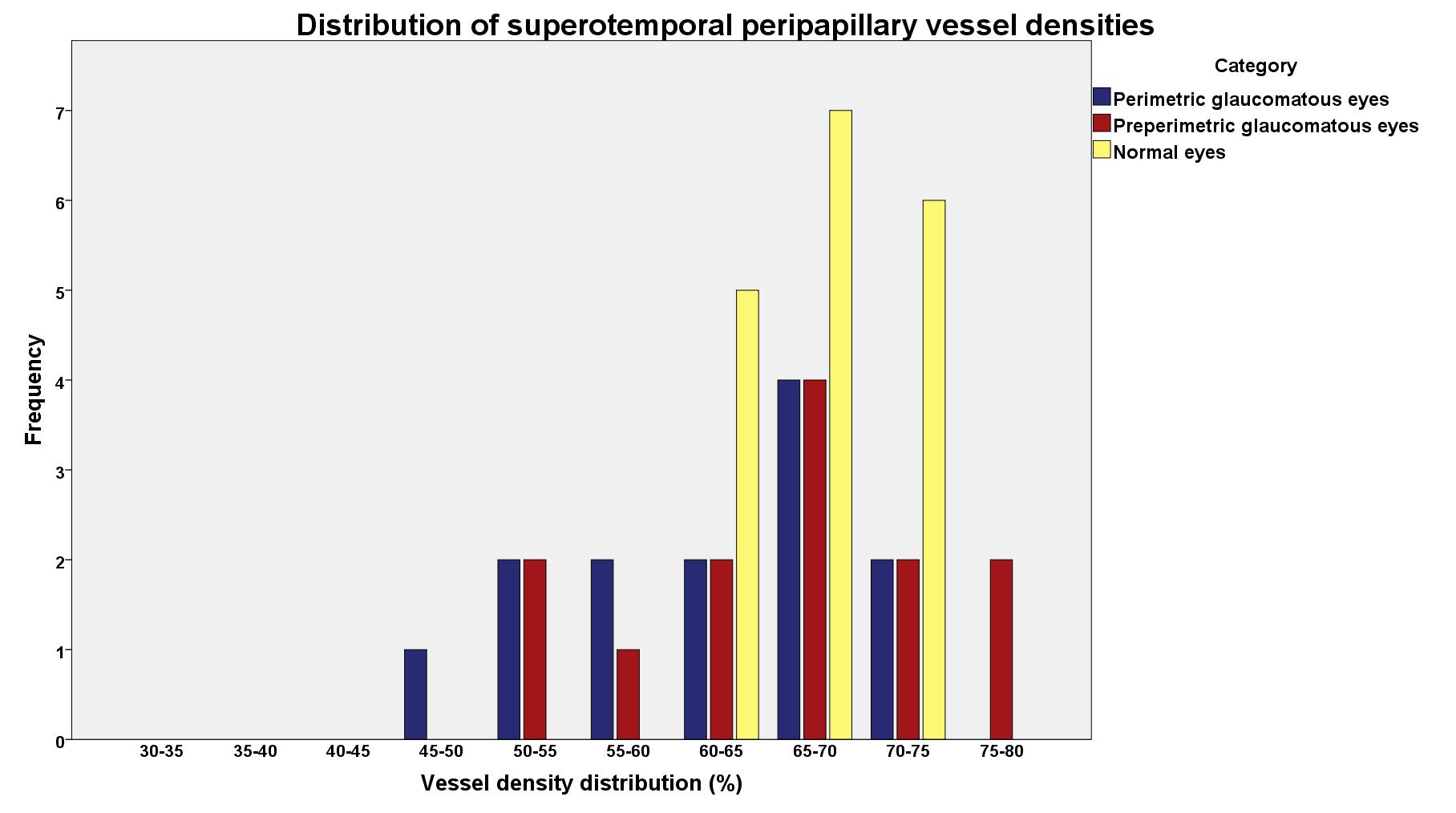

Supplement: S4 Fig — (JPG) [file pone.0184297.s004.jpg]
